# Identification of a Novel O-Conotoxin Reveals an Unusual and Potent Inhibitor of the Human α9α10 Nicotinic Acetylcholine Receptor

**DOI:** 10.3390/md15060170

**Published:** 2017-06-09

**Authors:** Shantong Jiang, Han-Shen Tae, Shaoqiong Xu, Xiaoxia Shao, David J. Adams, Chunguang Wang

**Affiliations:** 1 Department of Central Laboratory, Shanghai 10th People’s Hospital, School of Life Sciences and Technology, Tongji University, Shanghai 200092, China; jajst2010@163.com (S.J.); xsq1201@163.com (S.X.); shxx@tongji.edu.cn (X.S.); 2 Illawarra Health and Medical Research Institute (IHMRI), University of Wollongong, Wollongong, NSW 2522, Australia; hstae@uow.edu.au

**Keywords:** conotoxin, O-superfamily, nicotinic receptor, α9α10, linear peptide

## Abstract

Conotoxins are a pool of disulfide-rich peptide neurotoxins produced by cone snails for predation and defense. They are a rich reservoir of novel ligands for ion channels, neurotransmitter receptors and transporters in the nervous system. In this study, we identified a novel conotoxin component, O-conotoxin GeXXVIIA, from the venom of *Conus generalis*. The native form of this component is a disulfide-linked homodimer of a 5-Cys-containing peptide. Surprisingly, our electrophysiological studies showed that, in comparison to the folded monomers, the linear peptide of this toxin had the highest inhibitory activity at the human α9α10 nicotinic acetylcholine receptor (nAChR), with an IC_50_ of 16.2 ± 1.4 nM. The activities of the N-terminal and C-terminal halves of the linear toxin are markedly reduced compared with the full-length toxin, suggesting that the intact sequence is required to potently inhibit the hα9α10 nAChR. α9α10 nAChRs are expressed not only in the nervous system, but also in a variety of non-neuronal cells, such as cochlear hair cells, keratinocytes, epithelial and immune cells. A potent inhibitor of human α9α10 nAChRs, such as GeXXVIIA, would facilitate unraveling the functions of this nAChR subtype. Furthermore, this unusual nAChR inhibitor may lead to the development of novel α9α10 nAChR-targeting drugs.

## 1. Introduction

The predatory cone snails (genus *Conus*) use their venoms as a weapon for predation and defense [1]. Based on their prey, cone snails are classified into fish-, mollusk-, or worm-hunting species, implying that their predation venoms may be largely different. Under the defensive evolutionary pressure, all cone snails have also developed defensive venoms against predators [2,3]. No matter what the purpose is, their venoms are primarily composed of an array of disulfide-rich peptides (conotoxins) that target membrane ion channels and neurotransmitter receptors in the nervous system [4]. As the number of conotoxin components is estimated to be in excess of 200,000, they are considered as a reservoir for novel drug leads for neuroscience research and disease treatment [5,6,7]. For example, ω-conotoxin MVIIA isolated from *C. magus* acts as a selective N-type Ca^2+^ channel inhibitor and has been developed as an analgesic drug commercially known as Ziconotide or Prialt [8,9,10].

Conotoxins are produced initially as gene-encoded polypeptide precursors and then maturated through proteolytic cleavages, and often post-translational modifications. Based on the signal peptide sequences of conotoxin precursors, the highly variable conotoxins are grouped into different superfamilies [11]. However, conotoxins belonging to the same superfamily do not necessarily have the same cysteine framework or functional target. One remarkable example is the O-superfamily conotoxins that include Ca^2+^ channel-inhibiting ω-conotoxins, K^+^ channel-inhibiting κ-conotoxins, Na^+^ channel-modifying δ-conotoxins, and Na^+^ channel-inhibiting μO-conotoxins [12]. 

Nicotinic acetylcholine receptors (nAChRs) are key in transmitting signals in the central and peripheral nervous systems, opening a cation-selective pore upon the binding of acetylcholine (ACh) to the extracellular domain [13]. nAChRs exist as homo- or hetero-pentameric channels and 17 subunits have been recognized in vertebrate species (α1–α10, β1–β4, γ, δ, and ε). The skeletal muscle nAChR subtype is composed of α1, β1, δ, and ε (adult isoform) or γ (fetal isoform) subunits at 2:1:1:1 ratio. In contrast, neuronal nAChR subtypes are composed of only α and β subunits except for α7, α9, and α10 subunits, which can form functional receptors without the participation of β subunits. Each nAChR subtype has distinct tissue expression, pharmacological properties and physiological functions [14].

Although the heteromeric α9α10 nAChR is generally characterized as neuronal, it is also expressed in non-neuronal cells such as cochlear hair cells, keratinocytes, pituitary pars tuberalis, epithelial and immune cells [15,16]. Investigation of the physiological roles of the α9α10 subtype would be greatly facilitated by potent inhibitors of the human (h) α9α10 nAChR. In this study, we identified a novel 5 Cys-containing conotoxin from the venom of *Conus generalis*. The precursor sequence of this toxin demonstrated that it is a member of the O1-superfamily. Although the native form of this toxin is a disulfide-linked homodimer, surprisingly the linear peptide of this toxin is a potent inhibitor of hα9α10 nAChR, with an IC_50_ of 16.2 ± 1.4 nM. This novel nAChR inhibitor may lead to the development of novel α9α10 nAChR-targeting drugs.

## 2. Results

### 2.1. Purification and Identification of a Five-Cys-Containing Component from the Venom of *Conus generalis*

**Figure 1:**
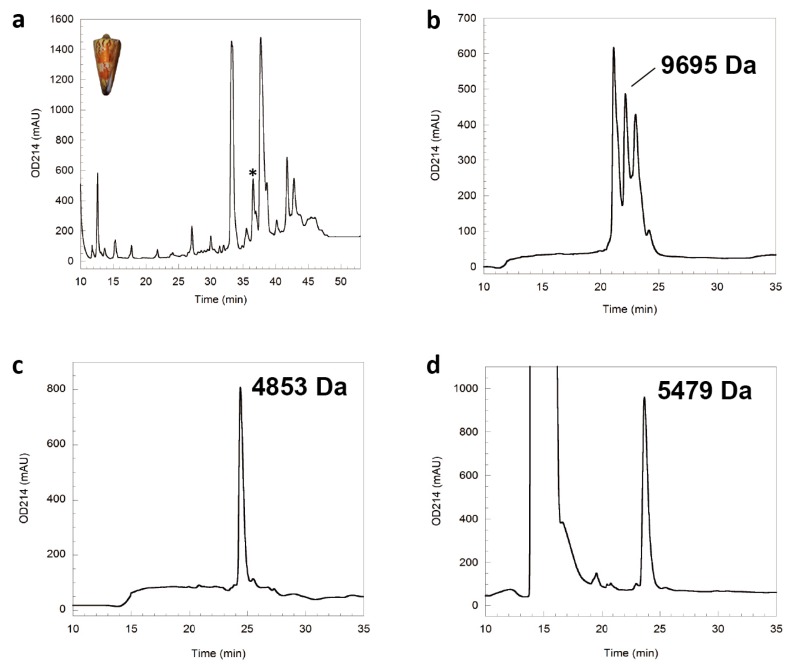
Purification and identification of O-conotoxin GeXXVIIA from *Conus generalis*. (**a**) Purification of crude venom extracted from *C. generalis* (shown in inset) on a ZORBAX C18 semi-preparative column. The asterisk indicates the fraction containing GeXXVIIA. The elution gradient is 0–50% Buffer B for 0–50 min with a flow rate of 0.5 mL/min. (**b**) Analytical scale purification of the GeXXVIIA-containing fraction from panel (**a**) on a C18 reverse-phase analytical column. The peak with a molecular mass of 9695 Da is that of GeXXVIIA. The elution gradient is 10–30% Buffer B for 0–10 min and 30–39% Buffer B for 10–37 min with a flow rate of 0.5 mL/min. (**c**) Purification of the reduced GeXXVIIA after being treated with DTT on a C18 reverse-phase analytical column. The elution gradient is 10–45% Buffer B for 0–35 min with a flow rate of 0.5 mL/min. (**d**) Purification of the GeXXVIIA peptide after being alkylated with NEM on a C18 reverse-phase analytical column. The elution gradient is the same as that of panel (**c**).

The separation of the *C. generalis* crude venom on a C18 semi-preparative reverse-phase HPLC column gave a series of peaks (Figure 1a). One minor peak, apart from two major peaks that have been studied previously [17,18], was focused in this study. A secondary separation using a C18 analytical column (Figure 1b) revealed that there are three components in this minor peak, with the second having a molecular mass of 9695 Da. Interestingly, reduction of this component with dithiothreitol (DTT) changed its molecular mass to 4853 Da (Figure 1c), indicating that this component is also a disulfide-linked homodimer, similar to αD-GeXXA [18]. Subsequently, alkylation with *N*-ethylmaleimide (NEM) of the reduced monomeric peptide increased the molecular mass to 5479 Da, which confirmed the presence of five Cys residues in this polypeptide chain (Figure 1d). The odd number of Cys residues is unique for conotoxins, which prompted us to investigate further.

### 2.2. Sequence Determination and cDNA Cloning

The reduced and alkylated monomeric peptide was first applied to Edman sequencing, which gave a partial N-terminal sequence of ALMSTGTNYRLP(T/K)(T/K)CRxSG, where x is an unidentified residue. Then, gene specific primers were designed, based on this N-terminal sequence, and 3’-RACE was carried out to obtain the 3’-partial cDNA sequence. The amino acid sequence deduced from the 3’-partial cDNA sequence was highly homologous to the sequence of O1-superfamily conotoxin Mik41 [19]. Therefore, we took advantage of the known cDNA sequence of Mik41 and designed an upstream primer to amplify the 5’-partial cDNA sequence of this toxin.

**Figure 2:**
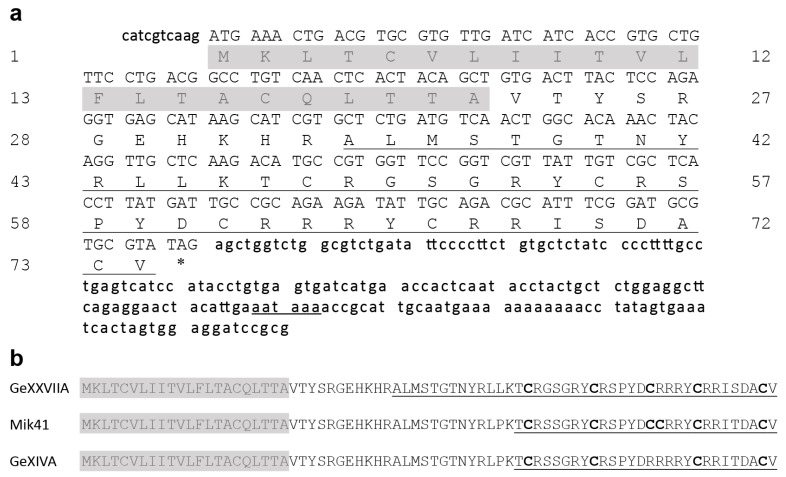
cDNA sequence of O-GeXXVIIA. (**a**) The cDNA sequence of GeXXVIIA and the cDNA-encoded precursor sequence. The coding region of cDNA is shown in capital letters. The signal peptide sequence is shadowed, and the mature peptide sequence is underlined. Between the signal sequence and mature toxin region is the pro-peptide region. * represents the stop codon. (**b**) Alignment of the precursor sequences of GeXXVIIA, Mik41 [19], and GeXIVA [20]. Mature toxin sequences are underlined with the Cys residues highlighted in bold.

The complete cDNA sequence of this toxin was obtained by overlapping the 3’-partial and 5’-partial cDNA sequences (Figure 2a). The cDNA-encoded precursor has a typical conotoxin organization of 22-residue signal peptide, 11-residue pro-peptide, and 41-residue mature toxin. The conserved signal peptide clearly indicates that this toxin belongs to the O1-superfamily. Additionally, the precursor sequence of this toxin is similar to two other O1-superfamily conotoxins, Mik41 and GeXIVA (Figure 2b) [19,20]. However, the new toxin has an additional 13 amino acid residues at the N-terminus of the mature peptide and 5 Cys residues compared to 6 for MiK41 and 4 for GeXIVA.

The cysteine framework of this conotoxin is different from other conotoxins that often contain even numbers of Cys residues. Therefore, we numbered this framework as XXVII and named this toxin GeXXVIIA. The molecular mass of the cDNA-deduced mature toxin, 4809.6 Da, is slightly different from the mass of the reduced natural toxin, which is likely due to sequence polymorphism or post-translational modifications.

### 2.3. Oxidative Refolding of GeXXVIIA

**Figure 3:**
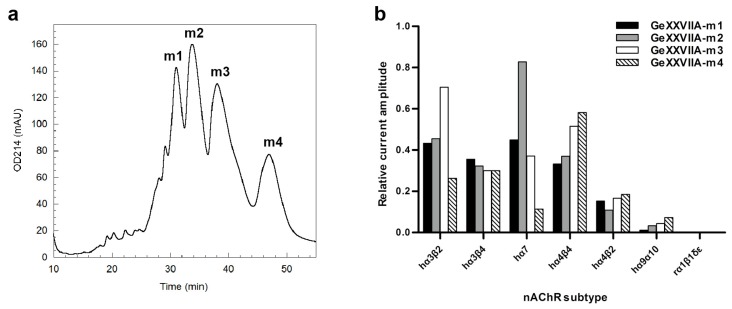
Preparation and nAChR-inhibitory activities of the monomeric isomers of GeXXVIIA. (**a**) Separation of four GeXXVIIA monomeric isomers (m1, m2, m3, and m4) refolded in vitro on a C18 reverse-phase analytical column. The elution is isocratic at 22% Buffer B with a flow rate of 0.5 mL/min. The molecular masses of these four isomers are the same, 4807 Da, indicating that two disulfide bonds are formed in each isomer. (**b**) The inhibition of 5 μM GeXXVIIA-m1, -m2, -m3, and -m4 on ACh-evoked peak current amplitude mediated by hα3β2, hα3β4, hα7, hα4β4, hα4β2, hα9α10, and rodent (r) α1β1εδ nAChRs (*n* = 1 oocyte for each nAChR subtype).

The unique sequence of GeXXVIIA motivated us to investigate its biological activity. Due to the low amount of the native GeXXVIIA, we sought to chemically synthesize the linear peptide of GeXXVIIA and then refold it in vitro. However, the regular air oxidation and glutathione oxidation conditions failed to produce a peak with the expected molecular mass. We managed to have two disulfide bonds formed in each peptide only in the condition of 400 mM arginine, with DTT and oxidized DTT as reducing-oxidizing pair reagents, and four different isomers with the same molecular mass were formed (Figure 3a). No dimeric product was observed, probably because the formation of the inter- and intra-chain disulfide bonds would require different oxidizing potential.

### 2.4. Inhibitory Effects of GeXXVIIA Monomeric Isomers on nAChRs

It has been reported previously that GeXIVA, which has similar sequence to the C-terminal sequence of GeXXVIIA, inhibits ACh-evoked current of nAChRs in a structure-insensitive manner [20]. Therefore, we tested the four monomeric isomers of GeXXVIIA (GeXXVIIA-m1 to -m4) at various nAChR subtypes heterologously expressed in *Xenopus laevis* oocytes, initially at 5 μM. Surprisingly, all GeXXVIIA isomers inhibited nAChR-mediated currents. Although the inhibition at hα3β2, α3β4, α7, and α4β4 nAChR subtypes was modest, these monomers all potently inhibited rodent (r) α1β1εδ and hα9α10 nAChR subtypes (Figure 3b).

### 2.5. Inhibition Activity of the Linear GeXXVIIA and Its Two Fragments

**Figure 4:**
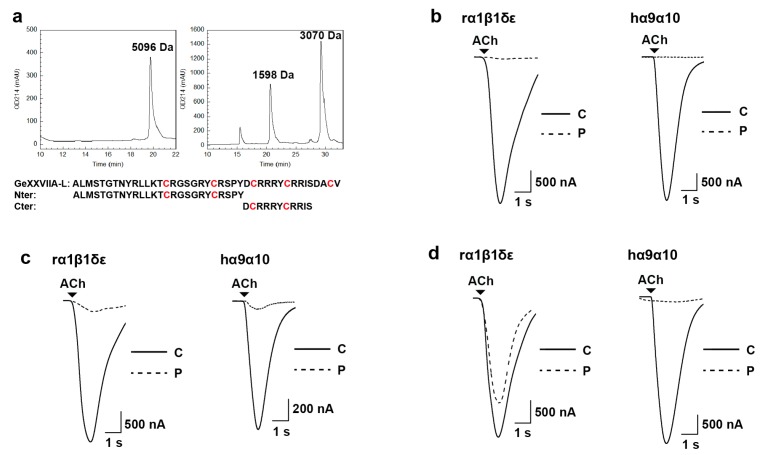
Preparation and nAChR-inhibitory activities of the linear GeXXVIIA and its N- and C-terminal fragments. (**a**) Left panel: Preparation of the linear peptide of GeXXVIIA (GeXXVIIA-L) alkylated with IAA on a C18 reverse-phase analytical column. Its molecular mass is 5096 Da. The elution gradient is 15–37% Buffer B for 0–22 min with a flow rate of 0.5 mL/min. Right panel: Two fragments were obtained after the digestion of GeXXVIIA-L by Asp-N protease. The elution gradient is 5–38% acetonitrile for 0–33 min with a flow rate of 0.5 mL/min. The N-terminal (Nter) and C-terminal (Cter) fragments of GeXXVIIA-L have a molecular mass of 3070 Da and 1598 Da, respectively. Below are the sequences of GeXXVIIA-L, Nter and Cter peptides, with the IAA-blocked Cys residues shown in red. (**b**–**d**) Superimposed ACh-evoked currents mediated by rα1β1εδ and hα9α10 nAChRs in the absence (**C**, control) and the presence of 5 μM different GeXXVIIA peptides (P). Arrows (▼) indicate ACh application. The peptides are GeXXVIIA-L (**b**), GeXXVIIA-L-Nter (**c**), and GeXXVIIA-L-Cter (**d**).

The results above demonstrated that the inhibitory activity of GeXXVIIA on different nAChR subtypes was not greatly affected by the different disulfide linkages in these isomers, similar to that of GeXIVA [20]. We then prepared a disulfide-free linear peptide of GeXXVIIA by modifying the Cys residues with iodoacetamide (IAA) (Figure 4a, left panel), and tested its activity at the two preferred subtypes, rα1β1εδ and hα9α10 nAChRs. Remarkably, the same concentration (5 μM) of the linear GeXXVIIA (GeXXVIIA-L) retained potent inhibitory activity on the ACh-evoked currents of these two nAChR subtypes (Figure 4b), which further highlighted that the folding of the peptide is not required for the interaction with nAChRs. This result also raised the possibility that the binding of the linear GeXXVIIA peptide with nAChR is only dependent on the sequence of the peptide.

In order to narrow down the critical sequence of GeXXVIIA-L responsible for the nAChR-inhibitory activity, we took advantage of the presence of the only two Asp residues in this toxin, and digested the linear GeXXVIIA with Asp-N protease into GeXXVIIA-L-Nter and GeXXVIIA-L-Cter fragments (Figure 4a). At 5 μM, the GeXXVIIA-L-Nter fragment strongly inhibited both rα1β1εδ and hα9α10 nAChR subtypes (Figure 4c), whereas the GeXXVIIA-L-Cter inhibited more strongly the hα9α10 nAChR (>90% inhibition) than the rα1β1εδ subtype (~30% inhibition) (Figure 4d). These results suggest that the N-terminal part of GeXXVIIA-L retains more nAChR inhibition activity than the C-terminal part, at this high concentration at least.

### 2.6. IC_50_ Values of the GeXXVIIA-L and GeXXVIIA-L-Nter

**Figure 5:**
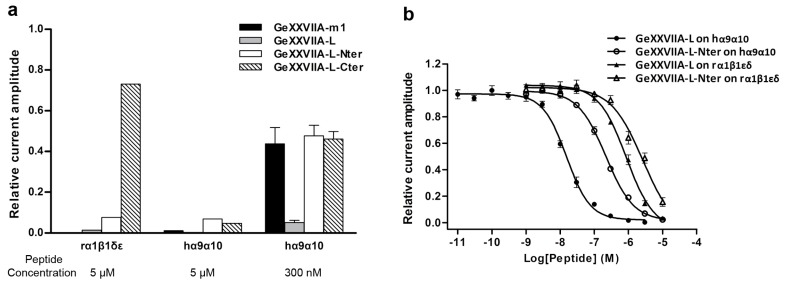
Antagonist potencies of GeXXVIIA-L and GeXXVIIA-L-Nter at the rodent α1β1εδ and human α9α10 nAChRs. (**a**) Inhibition of ACh-evoked peak current amplitude mediated by rα1β1εδ and hα9α10 nAChRs by GeXXVIIA-m1, GeXXVIIA-L, GeXXVIIA-L-Nter, and GeXXVIIA-L-Cter at 5 μM (*n* = 1 oocyte) and 300 nM (*n* = 3–7 oocytes) peptide concentrations. Error bars indicate SEM. (**b**) Concentration-response curves obtained for GeXXVIIA-L and GeXXVIIA-L-Nter inhibition of rα1β1εδ and hα9α10 nAChRs expressed in *Xenopus laevis* oocytes. Full-length GeXXVIIA-L exhibits potent inhibitory activity at hα9α10 receptors with an IC_50_ of 16.2 ± 1.4 nM (*n* = 4–8 oocytes for each data point).

At 5 μM, GeXXVIIA-L, GeXXVIIA-L-Nter, and -Cter fragments inhibited hα9α10 nAChR by >90% (Figure 4b–d), whereas at 300 nM, their inhibition levels diverged: the full-length linear GeXXVIIA still strongly inhibited hα9α10 nAChR (>90%), but the two fragments produced only ~55% inhibition (Figure 5a). Interestingly, when the folded monomer GeXXVIIA-m1 was tested at 300 nM as a reference, it gave a similar degree of inhibition as the two fragments of the linear peptide, but weaker than the full-length GeXXVIIA-L. Comparison between the inhibition levels of GeXXVIIA-m1 and GeXXVIIA-L implies that the disulfide linkage in the peptide decreased the inhibitory activity, on hα9α10 nAChR at least.

To obtain the quantitative potencies of the linear GeXXVIIA and GeXXVIIA-L-Nter, we measured their IC_50_ values at rα1β1εδ and hα9α10 nAChRs (Figure 5b). At rα1β1εδ nAChR, the full-length GeXXVIIA-L peptide gave an IC_50_ value of 774 ± 48 nM, whereas the GeXXVIIA-L-Nter fragment had an IC_50_ of 2.19 ± 0.24 μM, only about three-fold higher than GeXXVIIA-L, suggesting that the C-terminal part of the peptide does not contribute much to the interaction with this nAChR subtype. Unexpectedly, GeXXVIIA-L was highly potent at hα9α10 nAChR, with an IC_50_ of 16.2 ± 1.4 nM. Moreover, the potency of GeXXVIIA-L-Nter at this subtype was 14-fold weaker, with an IC_50_ of 222.5 ± 14.0 nM. The more dramatic difference between the full-length GeXXVIIA-L and GeXXVIIA-L-Nter suggests that the C-terminal part participates in the interaction with hα9α10 nAChR more than with rα1β1εδ nAChR.

### 2.7. Mechanism of Action of GeXXVIIA-L at hα9α10 nAChR

The high potency and the unique working conformation (linear peptide) of GeXXVIIA at hα9α10 nAChR encouraged us to explore its mechanism of action. To ascertain whether it binds to the orthosteric ACh binding site, we determined GeXXVIIA-L inhibition (applied at IC_50_ of 16.2 nM) of the hα9α10 nAChR-mediated currents evoked by different ACh concentrations. The ACh-evoked current amplitude was inhibited by 43.6 ± 1.4% (*n* = 10) when evoked with 6 µM ACh, 46.9 ± 2.4% (*n* = 11) when evoked with 30 µM ACh and 46.7 ± 2.9% (*n* = 9) when evoked with 100 µM ACh. The insurmountable inhibition of hα9α10 nAChR by GeXXVIIA-L when raising the ACh concentration indicates that GeXXVIIA-L is a non-competitive antagonist at hα9α10 nAChR. Furthermore, GeXXVIIA-L (16.2 nM) inhibition of ACh-evoked currents at hα9α10 nAChR was similar at all membrane potentials tested (45.6 ± 7.3% at −120 mV (*n* = 5), 44.9 ± 5.6% at −80 mV (*n* = 9), and 45.6 ± 8.1% at −40 mV (*n* = 4)). These results indicate that GeXXVIIA-L inhibition of hα9α10 nAChR is voltage-independent, suggesting that the peptide does not enter the membrane electric field and is unlikely to be an open channel pore blocker.

## 3. Discussion

Conotoxins are well known for their structural and functional diversity. In this study, we identified a novel conotoxin GeXXVIIA from the South China Sea mollusk *C. generalis* and classified it as a member of the O1-superfamily conotoxins. However, the mature peptide sequence of GeXXVIIA contains five Cys residues, which is distinct from the cysteine framework VI/VII (C–C–CC–C–C) in most of the O-superfamily conotoxins [12]. Comparison with the cDNA sequence of Mik41 suggests that the cysteine framework of GeXXVIIA may well be the result of a point mutation that changes the codon of the fourth Cys (TGC) in Mik41 into a codon of Arg (CGC) in GeXXVIIA [19]. The random point mutation in conotoxin genes has been found to be one of the mechanisms for conotoxin diversity [21]. Due to this unique cysteine framework, we propose that GeXXVIIA represents a new branch of the O1-superfamily.

It is uncommon for conotoxins to have odd number of Cys residues, as conotoxins are often disulfide-linked monomers, whereas an uneven number of Cys residues suggests the existence of interchain disulfide bond. Other conotoxins containing an odd number of Cys residues include TxXIIIA (with five Cys residues in the short sequence TSDCCFYNCCC) and the con-ikot-ikot (with 13 Cys residues in the mature sequence) [22,23]. Both of them form disulfide-linked homodimers, as believed for the native GeXXVIIA. However, the elucidation of the dimerization linkage remains a challenge. So far, the linkage is known only for two dimeric conotoxins, con-ikot-ikot and αD-GeXXA, by X-ray structure determination in both cases [18,24]. However, the very low abundance of the natural dimeric GeXXVIIA is a major obstacle for the structural study of this toxin.

Following the report of GeXIVA [20], we discovered that the monomeric GeXXVIIA has nAChR-inhibitory activity. Furthermore, different disulfide linkages were found not to change much the nAChR-inhibitory activity of GeXXVIIA (Figure 3), which is consistent with the properties of GeXIVA [20]. As the predicted sequence of GeXIVA differs from GeXXVIIA sequence mainly by the lack of N-terminal 13 residues, it appears that the basal nAChR-inhibitory activity of GeXXVIIA is mediated by the C-terminal part analogous to GeXIVA. However, quantitative comparison of the toxin activities suggests that the existence of the N-terminal extension in GeXXVIIA enhances the potency and specificity on hα9α10 nAChR.

The α9α10 subtype nAChRs were originally characterized in cochlear hair cells where they function in mediating synaptic transmission between the olivocochlear system and auditory hair cells of the cochlea [25,26,27]. Other studies reported that α9α10 nAChRs are expressed in different tissues, including dorsal root ganglia, skin, lymphocyte, sperm, and immune cells [28,29,30,31,32]. Potent α9α10-specific antagonizing conotoxins, such as α-RgIA, α-Vc1.1 and the bead isomer of GeXIVA, have been shown to provide long lasting relief in rat models of neuropathic pain, although the underlying mechanism remains controversial [20,33,34,35,36,37,38,39]. Recent studies have also shown that the α9 subunit is over-expressed in breast tumors [40]. Therefore, considering the physiological importance of hα9α10 nAChR, ligands with high potency on this nAChR subtype would be of great potential. However, the development of hα9α10 nAChR-selective conotoxins is hindered by the fact that most of the α9α10 subtype-specific conotoxins have only been studied on the rat nAChR subtype, examples including α-RgIA, α-Vc1.1, αS-GVIIIA, and αO-GeXIVA (Table 1). Prior to this study, only αD-GeXXA (IC_50_ = 28 nM) [18] and GeXIVA bead isomer (IC_50_ = 20 nM) [41] showed high potency at the hα9α10 subtype. The novel conotoxin GeXXVIIA identified in this study has a similarly high potency at the hα9α10 subtype (IC_50_ = 16.2 nM). However, unlike the conotoxins discussed above, in which their potency is dependent on the disulfide folding, GeXXVIIA is a more potent inhibitor as a linear peptide. This unique property not only facilitates the synthesis and use of this toxin, but also suggests that GeXXVIIA has an unusual nAChR-inhibition mechanism. Indeed, the comparable inhibition by GeXXVIIA-L tested with different ACh concentrations or at different membrane potentials demonstrates that GeXXVIIA-L is neither a competitive antagonist, nor a channel pore blocker of nAChR. Most likely, GeXXVIIA-L is a new class of nAChR allosteric inhibitor.

It is unusual among conotoxins that the linear GeXXVIIA is more potent than its folded counterparts (Figure 5a). This suggests that the interaction between GeXXVIIA and α9α10 nAChR is improved with an extended peptide of GeXXVIIA, most likely because the nAChR-binding sites in GeXXVIIA are distributed along the sequence of this toxin. This is probably why both the two halves of GeXXVIIA have diminished nAChR-inhibitory activities compared to the full-length toxin. In other words, both the two halves of linear GeXXVIIA cooperatively contribute to the high potency of this toxin. Given the unusual high content of basic residues (nine Arg and one Lys out of 41 residues) in the GeXXVIIA sequence (Figure 2), it is highly likely that the electrostatic interaction with the nAChR acidic residues plays a major role in binding. Consistent with this proposal, a strongly electronegative surface was identified in the extracellular vestibule from the crystal structural study of α4β2 nAChR [42]. Identification of the binding site of GeXXVIIA on α9α10 nAChR may furnish valuable information in understanding the gating mechanism of this receptor.

In summary, we have identified a novel O-superfamily conotoxin GeXXVIIA from the venom of *C. generalis*. We discovered that the linear peptide of GeXXVIIA can strongly inhibit the activity of the human α9α10 nAChR with an IC_50_ of 16.2 nM. Although nAChR may not be the target of the native GeXXVIIA toxin that is a disulfide-linked homodimer, the high potency of its linear peptide on hα9α10 nAChR per se is of great interest. These findings suggest an unusual inhibition mechanism on nAChR, which merits further investigation, both for the understanding of nAChR structure and function and for the development of novel nAChR inhibitors.

## 4. Materials and Methods 

### 4.1. Toxin Isolation and Purification

Specimens of *C. generalis* were collected from the South China Sea, near the coast of Sanya, Hainan Island, China. In order to extract the crude venom, the venom ducts were homogenized and suspended in 0.1% (*v*/*v*) trifuoroacetic acid (TFA). After centrifugation at 12,000× *g* at 4 °C for 15 min, the supernatant was collected and the pellet was stirred in 0.1% TFA for 30 min, then re-centrifuged. Precipitates were pooled and re-extracted successively with 0.1% TFA in 20, 30, 40 and 50% acetonitrile. Supernatants were combined, lyophilized, and stored at −20 °C.

To purify the conotoxins, the lyophilized crude venom was dissolved in Buffer A (0.1% TFA). After centrifugation, the supernatant was applied on a ZORBAX C18 semi-preparative column (9.4 × 250 mm, Agilent, Santa Clara, CA, USA) and eluted with a gradient of buffer B (0.1% TFA in 100% acetonitrile) using an Agilent 1100 HPLC system. Further purification was performed on a ZORBAX C18 analytical column (4.6 × 250 mm, Agilent, Santa Clara, CA, USA).

### 4.2. Toxin Characterization and N-Terminal Sequencing

The purified conotoxin component was dissolved in the reduction buffer of 100 mM Tris-HCl, pH 8.7 and 2 mM EDTA, and reduced with a 100-fold excess of dithiothreitol (DTT) at 37 °C for 1 h. The reduced peptide was purified on a ZORBAX C18 analytical column (4.6 × 250 mm, Agilent, Santa Clara, CA, USA). Thiol groups were alkylated with 10 mM *N*-ethylmaleimide (NEM) in the reduction buffer at 37 °C in the dark for 0.5 h. The modified peptide was purified by a C18 reverse-phase column on HPLC, and directly used for N-terminal sequencing on ABI 491A Procise^®^ Protein Sequencing System (Applied Biosystems, Foster City, CA, USA).

The mass spectrometry analyses of the native, reduced and S-alkylated toxins were performed on a Q-trap mass spectrometer (Applied Biosystems, Foster City, CA, USA), using the scan type of Enhanced MS. The apparatus was equipped with a TurboIonSpray source and operated in positive ionization mode.

### 4.3. cDNA Cloning

Total RNA was extracted from homogenized venom duct tissues using Trizol Reagent (Invitrogen, Carlsbad, CA, USA) according to the manufacturer’s protocol. Reverse transcription was carried out using a universal oligo dT-containing adapter primer 5’-GGC CAC GCG TCG ACT AGT AC (dT)_17_-3’ (Superscript II Kit, Invitrogen, Carlsbad, CA, USA). The resulting cDNA was used as a template for cDNA-cloning PCR.

Complete cDNA sequence of this toxin was obtained by overlapping the 3’- and 5’-partial cDNA sequences. The 3’-partial cDNA was first amplified through two rounds of PCR. The first PCR reaction was carried out using a degenerate oligonucleotide primer GSP1 (5’-GCNYTRATGTCNACNGGNACNAAYTAY-3’, Y: T/C, R: G/A, N: A/T/G/C), designed based on the N-terminal sequence (ALMSTGTNY-) and an outer primer provided by the 3’-RACE kit (Takara, Dalian, China). The second round of PCR amplification was carried out using the product of the first round PCR as the template by pairing the upstream primer GSP2 (5’-ATGTCNACNGGNACNAAYTAY-3’), designed based on the partial N-terminal sequence (-MSTGTNY-) with an inner primer provided by the kit. The ~250 bp PCR products were ligated into pGEM-T Easy vector (Promega, Madison, WI, USA) for sequencing.

Sequence comparison of the 3’-partial cDNA obtained above with other conotoxins showed that the mature peptide sequence of this toxin displays a high degree of homology to that of O-superfamily conotoxin Mik41 [19]. As the signal peptide of the O-superfamily is highly conserved, an upstream primer SP1 (5’-CATCGTCAAGATGAAACTGACG-3’) was designed based on the known cDNA sequence of Mik41. According to the 3’-partial sequence of GeXXVIIA, we designed a specific antisense primer GSP3 (5’-CACAGGTATGGATGACTCAGG’-3’) corresponding to the 3’-untranslated region. The 5’-partial cDNA of GeXXVIIA was amplified from the total cDNAs of *C. generalis* with these two primers and ligated into pGEM-T Easy vector (Promega, Madison, WI, USA) for sequencing.

### 4.4. Peptide Synthesis and Refolding

The linear peptide of GeXXVIIA, with all 5 Cys residues unprotected, was synthesized by the Chinese Peptide Company (Hangzhou, China). Oxidative folding of the purified linear peptide (20 μM) was performed at 4 °C for one week in a buffer comprising 50 mM Tris-HCl, pH 8.0, 400 mM arginine, 0.02 mM DTT, and 0.3 mM oxidized DTT. The oxidized products were purified on a ZORBAX C18 HPLC analytical column (4.6 × 250 mm, Agilent) with isocratic elution. Each monomer component was applied to a secondary HPLC purification.

### 4.5. Peptide Alkylation and Digestion by Protease Asp-N

The synthesized linear GeXXVIIA was alkylated with 10 mM iodoacetamide (IAA) in 100 mM Tris-HCl, pH 8.7, and 2 mM EDTA at 22 °C in the dark for 1 h. The alkylated linear peptide GeXXVIIA-L was purified on a ZORBAX C18 HPLC analytical column (4.6 × 250 mm, Agilent). The digestion of alkylated GeXXVIIA-L was carried out in 100 mM Tris-HCl, pH 8.5, using 3 μg/mL Asp-N (Sigma, St Louis, MO, USA) at 37 °C for 18 h. The digested products were separated on a ZORBAX C18 HPLC analytical column (4.6 × 250 mm, Agilent).

### 4.6. Oocyte Two-Electrode-Voltage Clamp Recordings

Oocyte preparation, RNA preparation, and expression of nAChR subunits in *Xenopus* oocytes were performed as described previously [12]. All procedures were approved by the University of Sydney Animal Ethics Committee. Plasmid constructs of rat (α1, β1, and δ), mouse (ε) and human (α3, α4, α7, α9, α10, β2, and β4) nAChR subunits were linearized for *in vitro* mRNA synthesis using mMessage mMachine transcription kit (AMBION, Forster City, CA, USA).

Stage V–VI *Xenopus laevis* oocytes were defolliculated with collagenase (Worthington Biochemical Corp., Lakewood, NJ, USA) at room temperature (20–25 °C) for 1 h in OR-2 solution containing (in mM) 82.5 NaCl, 2 KCl, 1 MgCl_2_, and 5 HEPES at pH 7.4. Oocytes were injected with 5 ng cRNA for hα3β2, α3β4, α4β2, α4β4, α7 or rodent α1β1δε nAChRs, and 35 ng cRNA for hα9α10 nAChR (concentration confirmed spectrophotometrically and by gel electrophoresis) using glass pipettes. Oocytes were incubated at 18 °C in sterile ND96 solution composed of (in mM) 96 NaCl, 2 KCl, 1 CaCl_2_, 1 MgCl_2_, and 5 HEPES at pH 7.4, supplemented with 5% fetal bovine serum, 50 mg/L gentamicin (GIBCO, Grand Island, NY, USA) and 10,000 U/mL penicillin-streptomycin (GIBCO, Grand Island, NY, USA).

Membrane currents were recorded from oocytes expressing nAChRs at room temperature, using a GeneClamp 500B amplifier and pClamp9 software interface (Molecular Devices, Sunnyvale, CA, USA) in a two-electrode voltage-clamp recording configuration (holding potential −80 mV). In a series of experiments, the effect of different membrane potentials (−120, −80, and −40 mV) on GeXXVIIA inhibition of hα9α10 nAChRs was tested. Voltage-recording and current-injecting microelectrodes were pulled from GC150T-7.5 borosilicate glass (Harvard Apparatus Ltd., Holliston, MA, USA), giving tip resistances of 0.3–1.5 MΩ when filled with 3 M KCl. Oocytes were perfused with ND96 solution at a rate of 2 mL/min. Oocytes expressing hα9α10 nAChRs were incubated in 100 µM BAPTA-AM ~3 h before recording and perfused with ND115 solution containing (in mM): 115 NaCl, 2.5 KCl, 1.8 CaCl_2_, and 10 HEPES at pH 7.4.

Initially, oocytes were briefly washed with bath solution (ND96/ND115) followed by three applications of acetylcholine (ACh) at half-maximal effective concentration (EC_50_) of 6 μM for hα3β2, hα9α10 and hα4β4 nAChRs, 300 μM for hα3β4, 100 μM for hα7, 3 μM for hα4β2, and 1 μM for rα1β1εδ nAChRs. Washout with bath solution was done for 3 min between ACh applications. GeXXVIIA-L inhibition of hα9α10 nAChRs was also examined at different ACh concentrations as indicated. Oocytes were incubated with peptides for 5 min with the perfusion system turned off, followed by co-application of ACh and peptide with flowing bath solution. All peptide solutions were prepared in ND96/ND115 + 0.1% bovine serum albumin. Peak current amplitude evoked by ACh was measured before and following incubation with peptide in order to determine the effect on specific nAChR subtype. Concentration-dependent response curves for antagonists were fitted by unweighted nonlinear regression to the following logistic equation:

E_x_ = E_max_ X^nH^/(X^nH^ + IC_50_^nH^), (1)

where E_x_ is the response, X is the antagonist concentration, E_max_ is the maximal response, n^H^ is the slope factor, and IC_50_ is the antagonist concentration giving half-maximal response. All electrophysiological data were pooled (*n* = 4–11 oocytes for each data point) and represented as means ± standard error of the mean (SEM). The IC_50_ was determined from the concentration-response curve and reported with error of the fit. Computation was performed using GraphPad Prism 5 (GraphPad Software, La Jolla, CA, USA).

## Figures and Tables

**Table 1:** Conotoxins with high potency on α9α10 nAChRs.

Super-Family	Toxin Name	*Conus* Species	Toxin Sequence	Species of α9α10 nAChR	IC_50_ (nM)	Reference
O	GeXXVIIA	*C. generalis*	ALMSTGTNYRLLKTCRGSGRYCRSPYDCRRRYCRRISDACV	*H. sapiens*	16.2	This study
O	GeXIVA	*C. generalis*	TCRSSGRYCRSPYDRRRRYCRRITDACV	*R. norvegicus*	4.6	[20]
				*H. sapiens*	20.3	[41]
A	RgIA	*C. regius*	GCCSDPRCRYRCR	*R. norvegicus*	1.5	[43]
				*H. sapiens*	494	
A	Vc1.1	*C. victoriae*	GCCSDPRCNYDHPEIC *	*R. norvegicus*	19	[33]
S	GVIIIB	*C. geographus*	SGSTCTCFTSTNCQGSCECLSPPGCYCSNNGIRQRGCSCTCPGT *	*R. norvegicus*	9.8	[44]
D	GeXXA	*C. generalis*	DVHRP CQSVRPGRVWGKCCLTRLCSTMCCARADCTCVYHTWRGHGCSCVM	*R. norvegicus*	1.2	[12]
				*H. sapiens*	28	

* C-terminal amidation.
